# A Metamaterial-like Structure Design Using Non-uniformly Distributed Dielectric and Conducting Strips to Boost the RF Field Distribution in 7 T MRI †

**DOI:** 10.3390/s24072250

**Published:** 2024-03-31

**Authors:** Santosh Kumar Maurya, Rita Schmidt

**Affiliations:** 1 Department of Brain Sciences, Weizmann Institute of Science, Rehovot 7610001, Israel; santosh-kumar.maurya@weizmann.ac.il; 2 The Azrieli National Institute for Human Brain Imaging and Research, Weizmann Institute of Science, Rehovot 7610001, Israel

**Keywords:** metamaterial-based design, magnetic resonance imaging, ultra-high field, non-uniform distribution

## Abstract

Metamaterial-based designs in ultra-high field (≥7 T) MRI have the promise of increasing the local magnetic resonance imaging (MRI) signal and potentially even the global efficiency of both the radiofrequency (RF) transmit and receive resonators. A recently proposed metamaterial-like structure—comprised of a high-permittivity dielectric material and a set of evenly distributed copper strips—indeed resulted in a local increase in RF transmission. Here, we demonstrate that *non-uniform* designs of this metamaterial-like structure can be used to boost the ultimate RF field distribution. A non-uniform *dielectric* distribution can yield longer electric dipoles, thus extending the RF transmit field coverage. A non-uniform distribution of *conducting strips* enables the tailoring of the local electric field hot spots, where a concave distribution resulted in lower power deposition. Simulations of the brain and calf regions using our new metamaterial-like design, which combines non-uniform distributions of both the dielectric and conducting strips, revealed a 1.4-fold increase in the RF field coverage compared to the uniform distribution, and a 1.5–2-fold increase in the transmit efficiency compared to the standard surface-coil.

## 1. Introduction

In clinical diagnosis, magnetic resonance imaging (MRI) provides rich multimodal information—from the anatomy, structure, and function of organs to blood vessel distribution and perfusion. The advent of ultra-high fields (≥7 T) in MRI [1,2,3,4] has led to improved imaging with far greater achievable spatial resolution and/or shorter scan durations. This improvement is due both to the higher magnetic field itself and to complementary significant advancements in MRI hardware [5]. The resulting improvement forms a milestone toward personalized medicine.

The move to higher magnetic fields, however, is accompanied by the challenge of increased Larmor frequency, which is associated with the magnetic field moving away from the magneto-static approximation [6]. At 7 T MRI, the radiofrequency (RF) field inhomogeneity arises from the relatively short wavelength (12–15 cm) in the tissue [7,8], making it comparable with the dimensions of the scanned object. Two major hurdles are increased RF field inhomogeneity and enhanced power deposition [9]. These challenges have mainly been addressed by developing multi-channel transmit coils [10] and optimizing their elements, which offers a unique opportunity to introduce new metamaterial-based designs [11,12,13,14].

Recent studies have examined the potential of the metamaterial-based designs to increase local MRI signal and efficiency of the RF transmit and receive resonators [15,16,17,18,19]. One such proposed metamaterial-like design was based on a hybrid structure comprised of a high dielectric material and a set of conducting strips [20,21]. Attaching the conducting strips to a high permittivity dielectric creates a set of electric dipoles, thus generating a structure with similar properties to a metamaterial with negative permittivity [22], such as those applied in optics. Another advantage is that the high dielectric material shortens the effective wavelength, which then allows straightforward implementations in the MRI practical RF frequencies. Thus, with the dielectric layer (which results in effective wavelength, λ, of ~12 cm), the distances between the strips were in the range of λ/6, which was small enough to generate material with properties different from these of the dielectric alone. This setup was able to increase the local RF transmit field for frequencies relevant to MRI, implemented and demonstrated in applications at 1.5 T [20] and at 7 T MRI [21]. This hybrid design further provides a thinner structure than one based solely on a dielectric layer. Although this type of structure is not a large periodic one, it corresponds to an approximation of small units (small compared to the wavelength) and provides a range of resonant modes.

The above structure, just as many other metamaterial-based designs, is made up of evenly distributed sub-units. In this study, we explore *non-uniform* distributions of the sub-units to boost the ultimate RF field distribution.

First, we explored the possibility of increasing the RF field coverage by prolonging the effective electric dipole. One approach to achieve this is to design a zigzag-shaped dipole, thus extending the actual strip length. This possibility has already been demonstrated in a dipole antenna [23] and in a metamaterial-based design [24]. Another option, explored in a recent study, is to use a non-uniform distribution of the dielectric to achieve a longer effective dipole in a dipole antenna. Here, we chose to explore a non-uniform dielectric design and its advantages for metamaterial-based structures [25]. As was shown in Ref. [26], increasing the permittivity toward the structure’s edges would increase the effective length of the electric dipoles, thus prolonging the RF field coverage along these dipoles. In our approach, parallel narrow dielectrics were connected via copper strips. A major advantage of such a design is a substantial reduction in the thickness over most of the structure’s area (i.e., copper strip regions). Thus, controlling the permittivity of the dielectric can be utilized to design more compact and flexible setups, which is important in a realistic, and often tight, coil-patient environment.

Second, we examined the RF field distribution of a set of non-uniformly distributed conducting strips. Both convex (denser in the center) and concave (denser at the edges) designs of the copper strips were studied, as well as the effect on the RF magnetic and electric fields. The hypothesis here is that a convex design will condense the intensity of the magnetic field, while a concave one will disperse it. The effect of the strips’ density in uniform and non-uniform distribution was investigated. Controlling the distribution as a principal property of the structure can offer additional control over the resulting hot spots in the electric field and thus affect the final power deposition, which is essential in MRI applications.

A final structure, which combined a non-uniform dielectric and a non-uniform distribution of the conducting strips, was added to an MRI setup in full 3D EM simulations to examine its benefits and to validate the results in a measurement setup.

## 2. Materials and Methods

### 2.1. Characterization of the Non-uniform Dielectric Distribution

**Figure 1:**
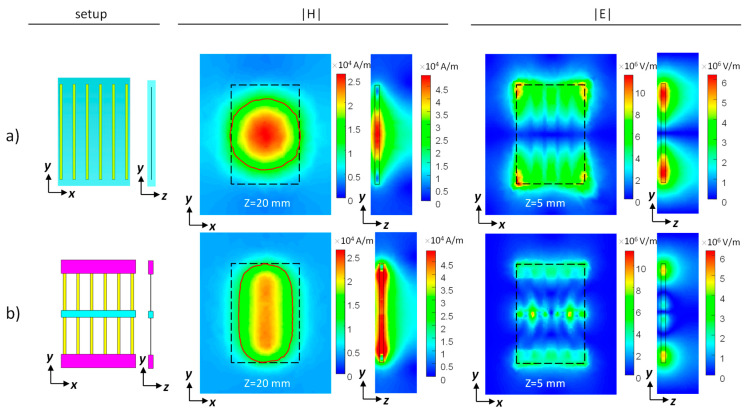
Hybrid metamaterial-like structures with a uniform or non-uniform dielectric. (**a**) Uniform design. (**b**) Non-uniform design. Shown from left to right are the structure schematics and RF field images in two cross sections: the |H| field map in parallel to the structure plane located 20 mm from the structure’s center and in a plane perpendicular to the structure; the |E| field map at 5 mm from the structure and in a plane perpendicular to the structure. Dashed, black overlay shows the structure dimensions, red overlay shows a contour of half-maximum.

An eigenmode solver via CST Studio Suite 2019 (Dassault Systemes, Vélizy-Villacoublay, France) was used to characterize the required parameters of the sub-units in a structure while realizing a resonant mode at 298 MHz. The overall dimensions of the structure were chosen as 16 × 11 × 0.7 cm^3^—a setup suitable for a realistic MRI environment. The resonant mode of interest was the lowest transverse-electric (TE) mode since it provides H-field components that are perpendicular to the main static magnetic field, and it can produce deep penetration outside the structure. The structure with a non-uniformly distributed dielectric was comprised of three short bricks of dielectric layers (two with the same permittivity at the borders and one with a different permittivity at the center) connected via conducting strips. For comparison purposes, a structure with a uniform dielectric layer was also produced. Both setups were constructed while keeping the overall dimensions. See Figure 1 for the schematics of the setups.

The details of the two setups are:

1. A uniform dielectric with uniformly spaced copper strips: a dielectric layer with the relative permittivity (ε_r_) = 72 and six copper strips equidistantly spaced 20 mm apart.
2. A non-uniform dielectric with uniformly spaced copper strips made up of three dielectric sections: central one 10 mm wide with ε_r_ = 60 and 20 mm wide sections with ε_r_ = 252 at each edge. The six copper strips were equidistantly spaced 20 mm apart.

### 2.2. Characterization of the Non-uniform Conductors’ Distribution

**Figure 2:**
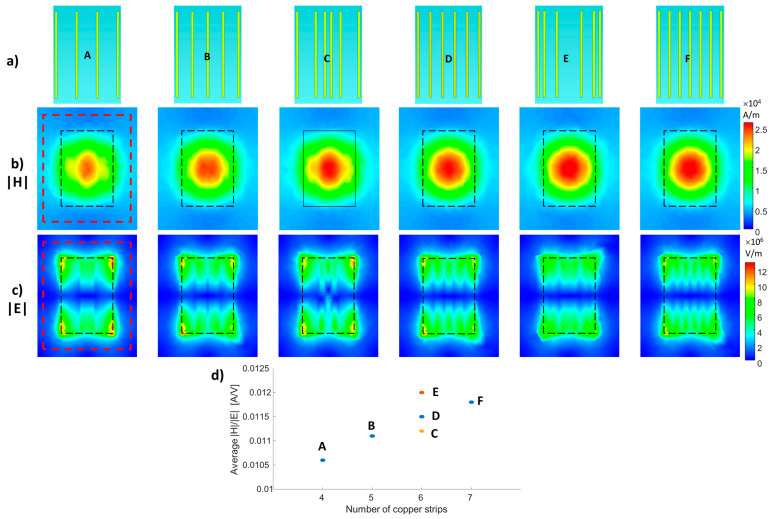
Different distributions of conducting strips. (**a**) Structure schematics. (**b**) |H| field map parallel to the structure plane located 20 mm from the structure’s center. (**c**) |E| field map at 5 mm from the structure. (**d**) Averaged |H|/E| ratio as a function of the different distributions. The A, B, D, and F configurations represent 4, 5, 6, and 7 uniformly distributed strips, respectively. The C and E configurations are of a convex and concave distribution of six strips, respectively. The black-dashed overlay shows the structure’s dimensions, and the red-dashed overlay shows the region of the averaged |H| and E| used to calculate the |H|/E| ratio.

To examine the effect of the conducting strips’ density, two sets of simulations were performed: (1) 4, 5, 6, or 7 strips with uniform distribution were added to a setup with an overall width of 11 cm and a uniform dielectric layer; and (2) a convex or concave distribution for the six-strip configuration. In the convex configuration, the five spacings between the six strips were (from left to right) 29, 15, 12, 15, and 29 mm; in the concave configuration, the five spacings between the six strips were (from left to right) 10, 20, 40, 20, and 10 mm. The distances were chosen such that the position of the strips at the boundary was kept the same. The schematics of the setups are shown in Figure 2.

**Figure 3:**
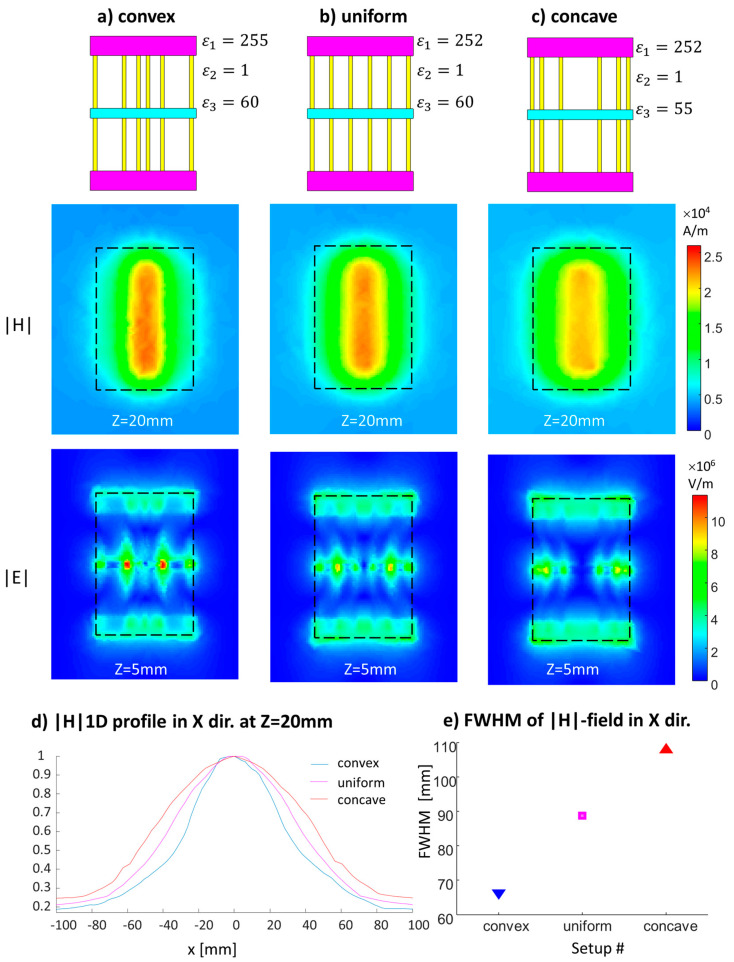
Combining a non-uniform dielectric distribution with a non-uniform distribution of the conducting strips. (**a**) Structure schematics of the convex, uniform, and concave configurations. (**b**) |H| field map parallel to the structure plane located 20 mm from the structure’s center. (**c**) the |E| field map at 5 mm from the structure. (**d**) Normalized 1D |H| profiled in the X direction at Z = 20 mm. (**e**) FWHM as a function of the configuration.

A set of simulations with the convex, uniform, and concave configurations was also performed for the six-strip configuration with the non-uniformly distributed dielectric. Small differences in the permittivity were required. See Figure 3 for the schematic and permittivity details.

To examine the H-field distribution outside the structure, an H-field map in a plane parallel to the structure at 20 mm from the structure center was used as a representing case of an imaging slice. To examine the E-field in proximity to the structure, where heating is likely to be greatest, an E-field map in a plane parallel to the structure at 5 mm from the structure center was used.

### 2.3. Full Setup Simulations

Three-dimensional EM simulations were performed using finite integration technique (FIT) software (CST Studio Suite 2019, Dassault Systemes, Vélizy-Villacoublay, France). To examine the structure incorporated in the MRI environment, two regions were examined—the lower extremity (calf region of interest) and the brain region of interest. The calf simulation comprised of the “Gustav” human model (mesh resolution 2 × 2 × 2 mm^3^). A structure placed near the head for local brain imaging enhancement was examined with the Duke model (mesh resolution 1 × 1 × 1 mm^3^). In this simulation, the metamaterial-like structure was curved to best fit the shape of the head.

All setups included a small ring-shaped surface-loop that was driven in 298 MHz. We compared the RF transmit field distribution with and without the metamaterial positioned below the calf region, using three metamaterial-like designs: (i) uniform distribution dielectric and conducting strips, (ii) non-uniform dielectric distribution with uniform conducting strips, and (iii) non-uniform dielectric distribution with a concave distribution of the conducting strips. In addition, to compare to a standard coil, a passive surface coil was designed with the same dimensions as the overall metamaterial-like dimensions of 16 × 11 cm^2^. This coil included two lumped elements tuning the coil to be resonant at 298 MHz.

To examine the effect of deforming the dielectric sections, two simulations of the non-uniform distributed dielectric structure were performed, each with a different shape of the dielectric sections, and compared to initial cuboid-shaped sections, keeping the same volume (see Supplementary Material Figure S1).

A simulation with a phantom was also performed, depicting the same electrical properties as those of the phantom used in the measurement setup. The phantom dimensions were 22 × 14 × 8 cm^3^, its relative permittivity (ε_r_) was 53, and conductivity (σ) was 0.3 S/m. These electrical properties mimic those in brain tissue.

The RF transmit field was calculated by the CST as a left circularly polarized field transverse to the main B_0_ magnetic field, commonly termed B_1_^+^ = (B_1x_ + B_1y_)/2. All B_1_^+^ maps were normalized to an accepted power of 1 Watt.

### 2.4. MRI Scanning and Implemented Structure

The metamaterial-like structure was experimentally realized by placing the copper strips on a very thin plastic substrate, which was placed in a pad sealed in a flexible plastic container. The ε_r_ = 252 dielectric layer was prepared with a BaTiO_3_–water suspension, and the ε_r_ = 52 layer consisted of a sucrose–water suspension. The phantom container was filled with a sucrose–water suppression with 52% sucrose and 0.5% NaCl to achieve ε_r_ = 53 and σ = 0.3 S/m.

The magnitude of the RF transmit field defines the excitation angle θ = γB_1_^+^τ, where γ is the gyromagnetic ratio and τ is the pulse duration, for a rectangular pulse, or an integral over B_1_^+^ for the more general case. The signal in the MRI images can be described as sin(θ)∙B_1_^−*^/√P, where B_1_^−^ is the receive RF field and P is the accepted power of the coil. To examine the effect on B_1_^+^, the B_1_^+^ maps with vendor’s B_1_ map pulse sequence were measured. Since we focus in this work on B_1_^+^ distribution, we term it B_1_ herein. All B_1_ maps were acquired with the same reference amplitude.

The examined structures were placed on top of the phantom and scanned in a 7T MRI (MAGNETOM Terra, Siemens Healthcare, Erlangen, Germany) with a 1Tx/32Rx Nova coil. Scans of the B_1_ map sequence were collected using a 20 × 20 cm^2^ FOV and spatial resolution 2.5 × 2.5 × 3.5 mm^3^, 25 slices.

## 3. Results

### 3.1. Non-uniform Dielectric and Conducting Strips’ Distribution

We compared the RF H- and E-field distributions of (1) a uniform dielectric (relative permittivity (ε_r_) = 72) with six uniformly spaced copper strips and (2) a non-uniform dielectric (a 10-mm-wide, ε_r_ = 60 central section flanked by 20-mm-wide, ε_r_ = 252 sections) with six uniformly spaced copper strips (Figure 1). The resultant H-field shows a prolonged H-field along the direction of the electric dipoles for the non-uniform distribution. H-field maps show a red contour overlay for half-maximum intensity. The half-maximum intensity coverage in the Y direction is 114 mm and 160 mm for a uniform and non-uniform distribution, respectively, achieving a 1.4-fold increase with the non-uniform distribution. However, the respective half-maximum coverage in the X direction is 1.33-fold smaller for the non-uniform distribution—114 mm and 86 mm for uniform and non-uniform distribution, respectively. The E-field peak values are detected close to the boundaries of both setups, while for the non-uniform distribution, local hot spots also appear at the center.

To examine the effect of the conducting strips’ density, we compared the H- and E-field distributions of setups with a uniform dielectric layer and 4, 5, 6, or 7 strips with uniform distribution, a non-uniform convex distribution with a six-strip configuration, and a non-uniform concave distribution with a six-strip configuration (Figure 2). Figure 2d shows the average |H|/|E| ratio as a function of strip density and distribution. Increasing the number of strips resulted in an increased |H|/|E| ratio. The convex and concave configuration show additional control of the average |H|/|E| ratio, with a lower value for the convex and a higher value for the concave configuration.

Figure 3 shows the convex, uniform, and concave configurations of the strips with a non-uniformly distributed dielectric. While the maximum of the H-field distribution is higher for the convex case, the concave configuration can produce wider coverage (Figure 3d shows a 1D normalized profile at the center). Figure 3e shows the estimated full-width-half-maximum (FWHM) in the X direction for the three setups. The concave vs. uniform configuration showed an increase of 22% in the FWHM in the X direction. An additional significant outcome is that the local hot spot peaks observed in the E-field are smaller in the concave configuration.

### 3.2. Full Setup Simulations and Measurements

A combination of both properties—the non-uniform distribution of the dielectric to prolong the RF B-field coverage and the non-uniform distribution of the conducting strips—can be utilized to better control the distribution of the RF transmit field and the resulting power deposition. To examine the potential for the MRI applications, we performed a set of simulations that included an RF coil and a virtual human model. Here, we examined the six-strip configuration with uniform and non-uniform dielectric distribution. The second configuration was tested with uniform and concave copper-strip configurations.

**Figure 4:**
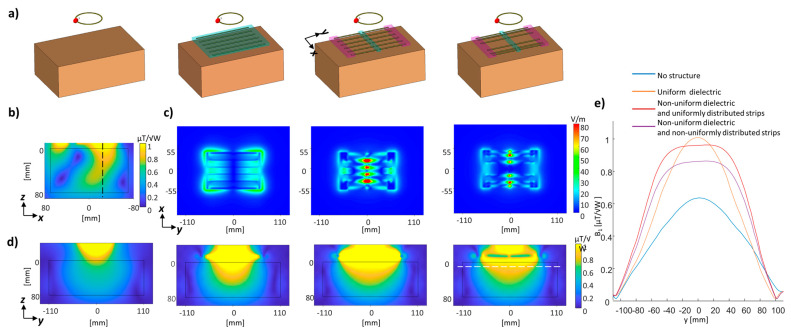
3D EM simulation with a phantom. (**a**) The setup. (**b**) The B_1_ map in the XZ plane with a dashed line that shows the YZ plane. (**c**) The E-field map in a cross-section of the structure. (**d**) The B_1_ map in the YZ plane (its location is shown as a black dashed line in (**b**). (**e**) 1D profile along the Y direction (see white dashed line).

A 3D EM simulation with a rectangular phantom showed similar distribution to that observed in the eigenmode solver, with reduced E-field local hot spots (Figure 4c) in the concave non-uniformly distributed strips compared to the uniform distribution (E-field peak of 108 V/m compared to 170 V/m, respectively). Figure 4b shows the B_1_ map in a plane perpendicular to the main static magnetic field (B_0_), revealing an asymmetric distribution around the center of the surface coil. This is an expected phenomenon at ultra-high fields (explained in Ref. [27]) which occurs due to interference patterns in high fields. Figure 4d shows the RF field distribution in the YZ plane (Y is along the direction of the conducting strips), and Figure 4e shows the B_1_ coverage along the Y direction. The increased coverage of the non-uniformly distributed dielectric is 150 mm, compared to 110 mm with the uniform dielectric (1.36-fold larger). The setup with the non-uniform conducting strips featured slightly lower maximal B_1_.

**Figure 5:**
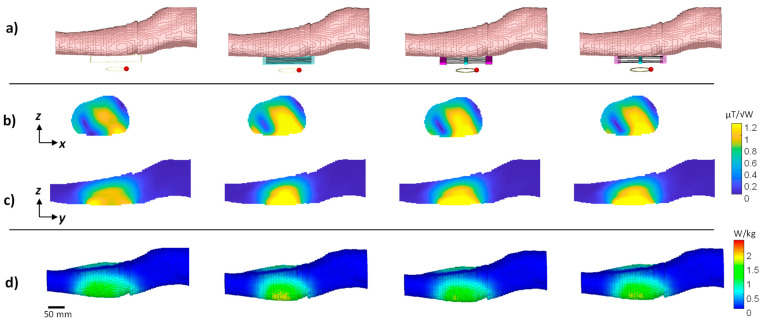
3D EM simulation in a calf region of interest. (**a**) The setup. (**b**,**c**) The B_1_ map in XZ and YZ planes. (**d**) 3D view of the SAR map.

Next, we performed a 3D EM simulation of a calf region of interest with a Gustav human model. Figure 5 shows the B_1_ maps in the two orthogonal planes perpendicular to the structure. Here, we also compared the metamaterial-like structures with a passive surface coil as a reference. The simulation in the calf region of interest showed a 1.9-, 1.68-, and 1.67-fold increase in the B_1_ peak compared to the passive surface-coil for a uniform dielectric, non-uniform dielectric, and combined non-uniform dielectric and conducting strips, respectively; the SAR increased by 18%, 15%, and 14%, respectively. Thus, the transmit efficiency (defined here as B_1_/√SAR_max_) for the uniform dielectric, non-uniform dielectric, and combined non-uniform dielectric and conducting strips increased by ×1.75, ×1.57, and ×1.56 compared to a passive surface-coil. Although the maximal efficiency was reached with the uniform dielectric, the B_1_ coverage along the dipoles direction increased 1.4-fold in the non-uniform dielectric configurations. In this example, a slight reduction (~1%) in the SAR was achieved with the non-uniform distribution of the conducting strips. The effect of deforming the dielectric sections on the B_1_ and SAR was examined with two curved setups, comparing to initial cuboid-shaped sections, while keeping the same volume. The different shapes did not have any effect on the B_1_ and had a negligible effect on the SAR (see Supplementary Material Figure S1).

**Figure 6:**
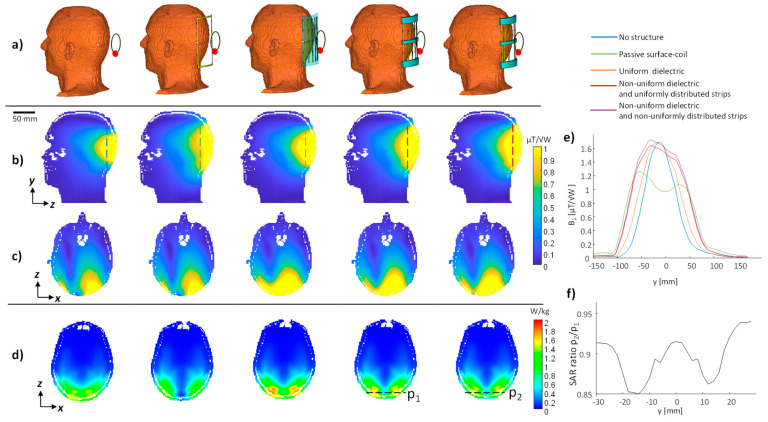
3D EM simulation in a brain as a region of interest. (**a**) The setup. (**b**) The B_1_ map in the YZ plane, (**c**) The B_1_ map in the XZ plane, (**d**) SAR map at a plane with a maximal SAR peak. (**e**) 1D profile along the red-dashed line in (**b**). (**f**) 1D profile of SAR along the black-dashed line in (**d**).

The simulation in the brain as a region of interest, with a structure added at the back of the head, compared three metamaterial-like structures and a passive surface-coil (Figure 6). Compared to the passive surface-coil, the B_1_ peak increased 1.99-, 1.99-, and 2.13-fold in the uniform dielectric, non-uniform dielectric, and combined non-uniform dielectric and conducting strips. The SAR deviated from the passive surface-coil by 9%, 1%, and −6%, while the transmit efficiency increased 1.9-, 1.98-, and 2.20-fold, respectively. The maximal SAR was reduced by 8% by the non-uniform copper strips’ distribution (compared to the uniform strips’ distribution). All SAR values are summarized in Table 1. Figure 6 shows a 1D plot of the local SAR that reaches a reduction of 15%. In another set of simulations, which involved an addition of the metamaterial-like structure near the left ear, the SAR reduction using the non-uniform conducting strips’ distribution (compared to uniform) was even more significant—a 14% reduction of the SAR (see Supplementary Material Figure S2 and Table 1).

**Figure 7:**
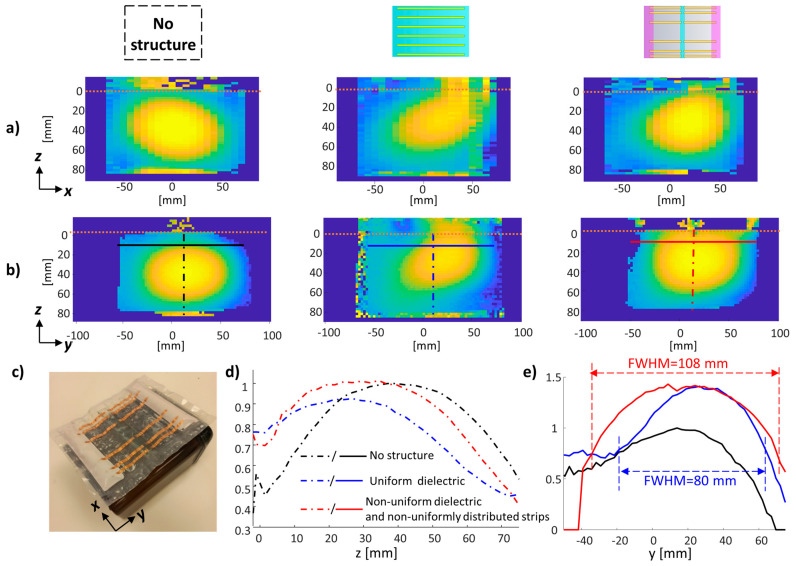
Measured B_1_ maps. (**a**) the B_1_ map in XZ plane. (**b**) B_1_ map in YZ plane. (**c**) Setup photo. (**d**,**e**) 1D profiles of B_1_ along Z and Y directions. B_1_ maps were acquired with the same reference amplitude and the maps were normalized to the maximal intensity of the scan without an added structure. The orange horizontal dotted line indicates the phantom edge. The B_1_ profiles in (**d**) (dot-dashed) and (**e**) (solid) are along the black, blue, and red lines shown in (**b**). Each color marks a different setup.

Finally, the uniform and combined non-uniform configurations were implemented and the B_1_ maps were measured in a 7T MRI with a phantom with brain-mimicking electric properties (Figure 7). The measured scans showed a 1.67- and 1.56-fold increase in B_1_ for uniform and non-uniform configurations, respectively. A similar increase of ×1.7 and ×1.55 for the uniform and non-uniform setups, respectively, was shown by a simulation (Figure 4). A 1.35-fold increase in the FWHM was measured, a result similar to the simulated 1.36-fold increase.

## 4. Discussion and Conclusions

MRI is one of the clinical applications that can greatly benefit from the development of metamaterial-based RF structure designs. Enhancing the RF field’s efficiency and reducing the power deposition are important goals to shorten the scan duration and increase the imaging resolution. Initial results with MRI-viable metamaterial-based designs are encouraging. However, the relatively large wavelength, on one hand, and the patient–environment-constricted dimensions, on the other, generate new complications compared to other EM fields with studied in-depth metamaterial structures. Understanding the principal properties that control and shape the RF field is central to the advancement of metamaterial designs in MRI. While most metamaterial designs are based on equally spaced sub-units, in this study, we examined how one can harness the spatial distribution of the sub-units to provide additional control over the resulting RF efficiency.

We were able to construct a non-uniform distributed dielectric with increased permittivity towards the structure’s edges that prolonged the RF transmit field coverage. In our design, the uniformly distributed dielectric was replaced by three narrow dielectrics with connecting copper strips regions. This strategy substantially reduced the thickness over most of the structure area, thereby reducing the total volume by ×2.2 and generating a flexible, compact structure that can be easily incorporated in an MRI setup. The eigenmode solver simulations showed that the FWHM of the H-field distribution is 1.4-fold longer for a structure with the same overall dimensions. Future optimization steps will entail building a targeted design per imaging region of interest in which the local E-field hot spots are controlled while considering the significant tissues in the targeted region of interest.

Another property that was analyzed by a set of eigenmode solver simulations is the density of the conducting strips with uniform and non-uniform (convex and concave) distributions. Increasing the density of the strips resulted in an increased |H|/|E| ratio. The convex configuration can be utilized to focus and increase the local H-field peak, while the concave distribution can be utilized to disperse the H-field distribution. Importantly, the strips’ distribution can be employed to control the hot spots of the electric field. It was observed that the convex configuration reached a higher H-field peak, but resulted in higher local E-field hot spots. This outcome is important when investigating the power deposition for a specific application. Depending on the application, convex or concave configurations can be of interest. Note that a non-uniform distribution of both the conducting strips and the dielectric was manually tailored under practical constraints of the setup, with a simple quadratic rule for the strips’ distances. Therefore, further studies to generalize and further improve the distribution should be performed.

3D EM full-setup simulations were performed to examine the effect on the RF B_1_ and the SAR. The simulations in the calf and brain as regions of interest demonstrated a 1.7–2.13-fold increase in the B_1_ peak, with the examined metamaterial-like setups compared to the surface-loop. The brain simulations also showed an SAR reduction of 8–14% while using non-uniform distribution of the strips compared to the uniform distribution. In all cases, the B_1_ coverage was 1.35–1.4-fold higher along the dipoles direction with the non-uniform dielectric compared to the uniform dielectric. The phantom measurements confirmed the increase in B_1_ peak and coverage.

In conclusion, this study provides new insights into a metamaterial-based design for MRI applications. We demonstrate the advantages of a non-uniform distribution of the dielectric and conducting strips, i.e., an increase in the RF field coverage and that ability to tailor the local E-field hot spots. While the current study demonstrates the utility of a metamaterial-based structure that is added passively to the driving coil, the structure can also serve as an alternative building block to the multi-channel RF coils. In the current implementation, a BaTiO_3_–water suspension was used, which has the advantage of a flexible cushion-like implementation, but the disadvantage of increased conductivity. In future designs, an artificial dielectric [28] can be utilized to replace the high-permittivity dielectric, further reducing the overall thickness of the setup.

## Figures and Tables

**Table 1:** Maximal local SAR [W/Kg] (averaged per 10 g) estimated in the 3D EM simulations with human models (in calf and brain as regions of interest).

Setup	Region of Interest		
	Calf	Brain, Structure Added at the Back of the Head	Brain, Structure Added Near the Left Ear
No added structure (only the driving surface-loop)	1.86	1.54	1.00
Added passive surface-coil of the same dimensions as the examined structures	1.69	1.61	1.18
Added structure with a uniform dielectric and distribution of the conducting strips	2.15	1.76	1.34
Added structure with a non-uniform dielectric and a uniform distribution of the conducting strips	1.94	1.63	1.40
Added structure with a non-uniform dielectric and distribution of the conducting strips	1.92	1.51	1.23

## Data Availability

Data are contained within the article.

## Supplementary Materials

The following supporting information can be downloaded at: https://www.mdpi.com/article/10.3390/s24072250/s1, Figure S1. 3D EM simulations that examined the effect of deforming the dielectric sections. (a) Three examined shapes—cuboid-shaped and two curved options, (b) the EM simulation setup in a calf as a region of interest with a structure added at the bottom. (c,d) The B_1_ map in XZ and YZ planes. The resonant frequency and the maximal SAR for each case is added at the bottom. Figure S2. 3D EM simulation in a brain as a region of interest with a structure added near the left ear. a, The setup. b, The B_1_ map in the YZ plane, c, The B_1_ map in the XZ plane, d, SAR map at a plane with a maximal SAR peak.
