# Supplementary figures and images for: A Metamaterial-like Structure Design Using Non-uniformly Distributed Dielectric and Conducting Strips to Boost the RF Field Distribution in 7 T MRI
Source: Sensors (Basel). 2024 Mar 31;24(7):2250. doi: 10.3390/s24072250 (PMC11014008; doi:10.3390/s24072250)

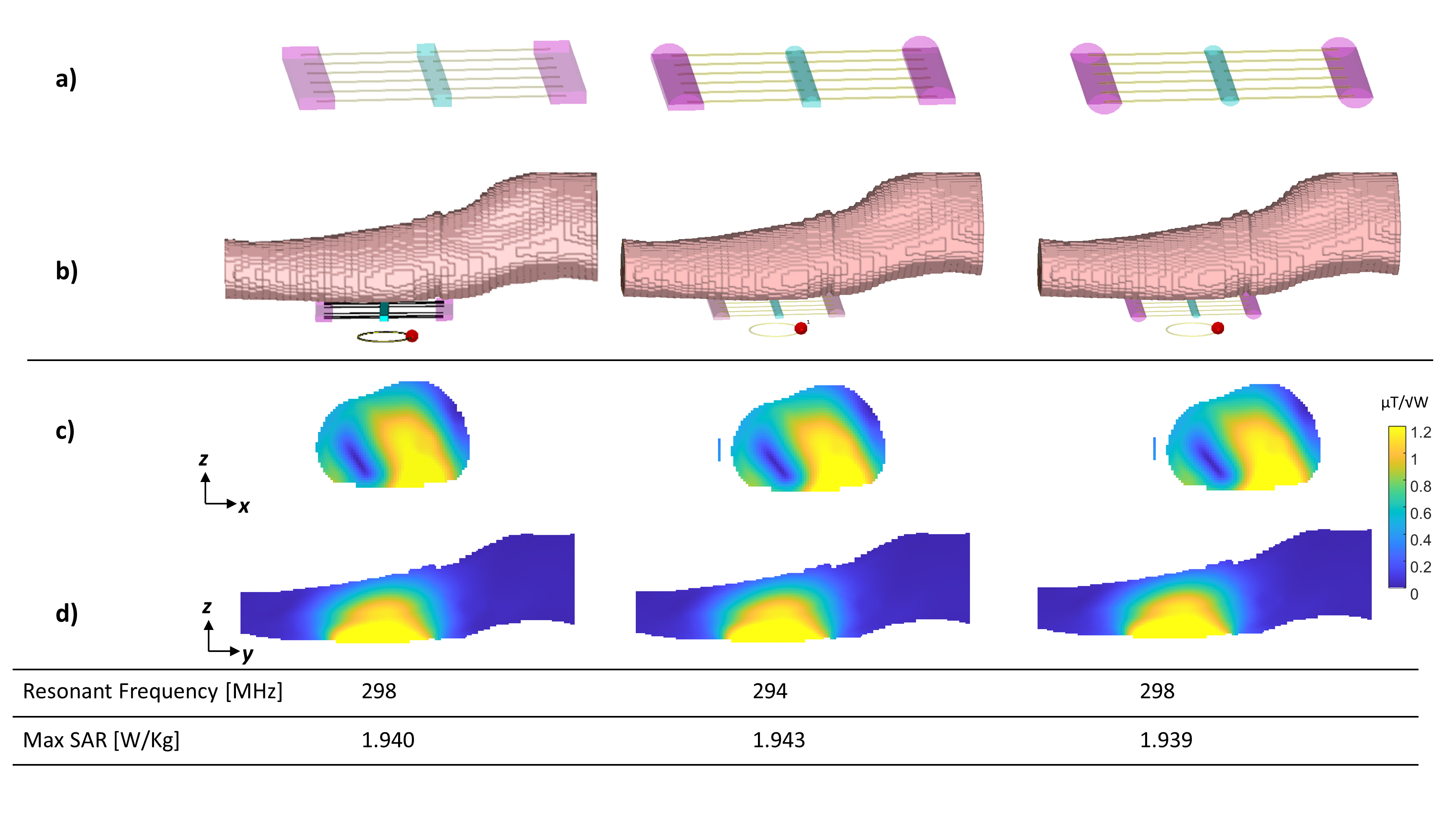

Supplement: Supplementary file 1 [file sensors-24-02250-s001.zip › FigureS1.tif]

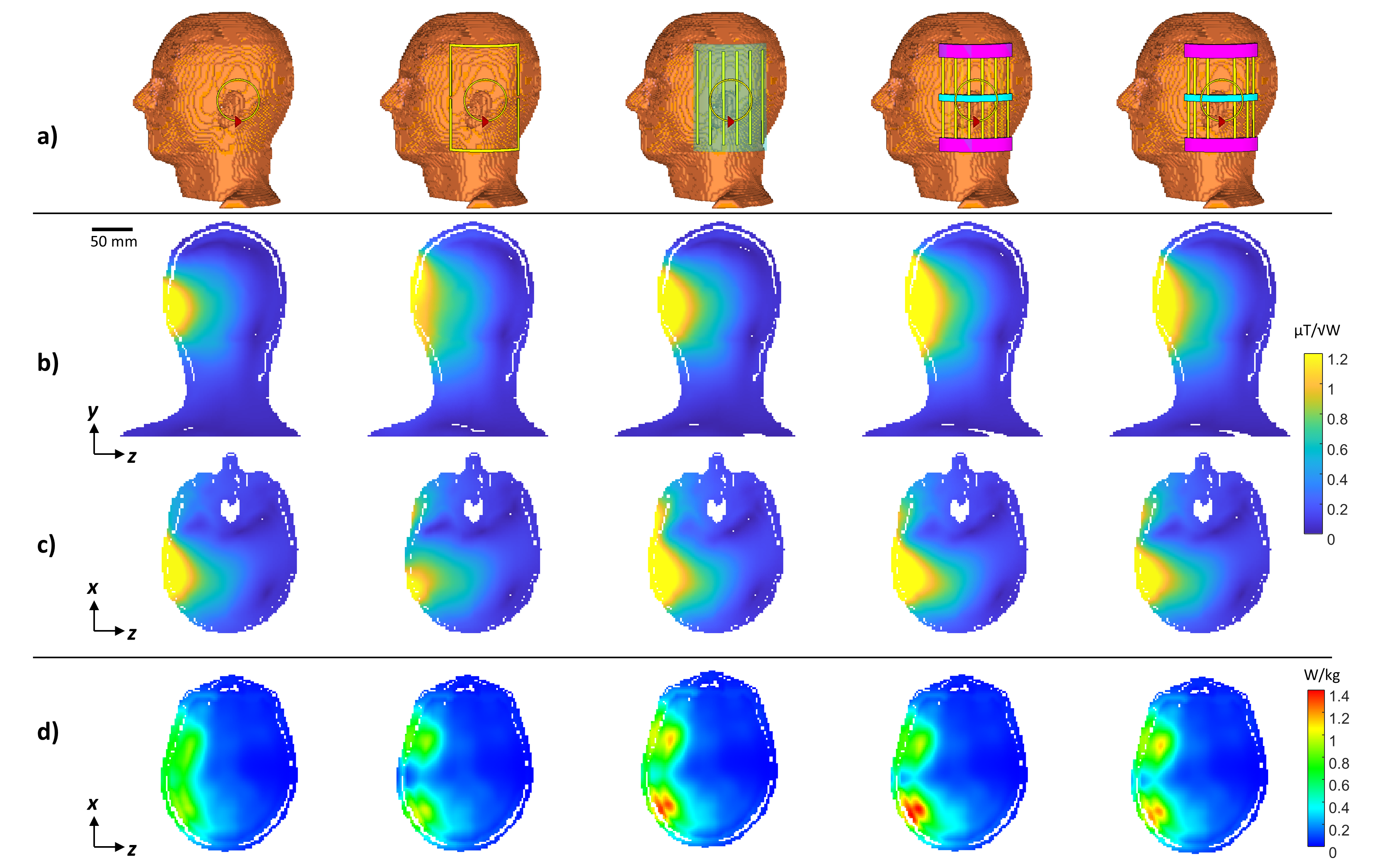

Supplement: Supplementary file 1 [file sensors-24-02250-s001.zip › FigureS2.tif]
